# Developing a theory of change – the importance of rich process data and authors’ insights into context, implementation and mechanisms

**DOI:** 10.1177/17579759241232387

**Published:** 2024-03-04

**Authors:** Helen Elizabeth Denise Burchett, Rebecca S. French, Sally Griffin, Málica de Melo, Joelma Joaquim Picardo, Dylan Kneale

**Affiliations:** 1 Department of Public Health, Environments & Society, Faculty of Public Health & Policy, London School of Hygiene & Tropical Medicine, London, UK; 2 International Centre for Reproductive Health: Mozambique, Mozambique; 3 EPPI-Centre, UCL Social Research Institute, University College London, London, UK

**Keywords:** adolescent, contraception, evaluation, evidence synthesis, process evaluation

## Abstract

**Background:**

Theories of change explaining how interventions work are increasingly important, yet the methods/data to develop these are less advanced than for evaluating effects.

**Methods:**

We conducted a systematic evidence synthesis to develop a theory of change for structural adolescent contraception interventions. We reflect on the utility of the information provided in evaluation reports.

**Findings/discussion:**

Few of the included evaluations presented their theory of change, or included rich, qualitative process data. Authors’ descriptions of context and implementation, typically in introduction and discussion sections, were very useful. These helped to understand the intervention’s context, how it was experienced and why or how it had the effect that it did. We recommend incorporating rich process evaluations into studies, and reporting contextual insights into the intervention’s development, implementation and experience. We also recommend including these data and insights within syntheses that aim to develop theories of change.

## Introduction

Outcome evaluations tell us whether an intervention is effective or not. However, it has been increasingly recognised that it is also important to understand *how* an intervention works (i.e. what the causal mechanisms are), to explain why some interventions are more or less effective than others, and to consider whether an intervention is likely to be applicable to a new context (1–3). To answer these questions, we also need to know how the intervention was implemented, what the context was and how it was experienced.

A theory of change sets out how an intervention is expected to lead to an outcome, describing the causal mechanisms and what contextual and other factors may affect their enactment (4). It is useful throughout the process of intervention development and evaluation, from helping to determine what the intervention should be and how it should be delivered, to shaping the evaluation and finally, refining the theory based on empirical evidence gathered through evaluation (5). However, despite increasing awareness of their importance, the methods and data required for developing and refining theories of change are not as advanced as those for evaluating effects (4). Furthermore, despite their utility across different stages of an intervention, they are more commonly used to inform intervention development, rather than empirically testing a theory of change (5). Therefore, while a theory of change is often included to inform or represent the design of an intervention or evidence synthesis, less attention is given to evaluating and updating this theory based on knowledge developed through the research/evaluation itself.

Theories of change can be useful at the individual study level, but can also be developed through synthesis of multiple studies (6). One approach is to use meta-ethnography to synthesise individual studies’ theories of change (7). However, studies’ reporting of theories of change are by no means universal and their prevalence varies between fields of research (7,8). In instances where insufficient studies have reported theories of change to allow synthesis, reviewers could draw on other data and information provided in the studies, for example, from process evaluations, to develop a theory of change for a body of evidence.

Calls have been made in recent decades for process evaluations to accompany outcome evaluations and the number of process evaluations published has increased (3,9). However, process evaluations often focus on quantitative data, reporting what took place, for whom, and/or measures of the perceived acceptability of the intervention, rather than exploring how an intervention was experienced, or how and why it had an effect (10,11). Such quantitative data can be relatively simple to collect and analyse, but lacks the richness to help understand an intervention’s causal mechanisms.

The question therefore remains, how to best conduct a synthesis that helps to understand interventions’ mechanisms of action, when this has not been set out within a study and when rich process data are lacking.

Standard evidence syntheses of effectiveness typically draw mainly on the research methods and findings sections of evaluation reports, extracting data on the intervention content, study design and results. In this short communication, we reflect on the data we found useful in a recent evidence synthesis undertaken to develop a theory of change.

## Materials and methods

We undertook a systematic evidence synthesis to develop a theory of change for structural interventions to enable adolescent contraceptive use (12). We wanted to understand how interventions were implemented and experienced to help explain their effect (or lack there of). We used a case-based approach, intervention component analysis (ICA), that drew on insights from all aspects of the included literature (13). ICA is an iterative method that involves extracting information about all aspects of the intervention and its evaluation (see Burchett *et al.* (12) for more details). The benefits of the method stem from its ability to draw insights from reports beyond those traditionally incorporated into reviews, particularly those related to the evaluation’s context and the intervention’s implementation – two factors that are understood to be of critical importance for developing theories of change. During and after the analysis, the team discussed and reflected on the types of data and insights that were most helpful in informing the development of our theory.

## Results/discussion

Only a minority of the included studies explicitly set out their theory of change and those that did rarely reflected on or refined it based on empirical data gathered in their evaluation. We found the most useful data for us were often from authors describing the context and interactions within it, typically found in introduction and discussion sections. For example, in an evaluation of a Bangladeshi intervention teaching computer skills to adolescent girls, the authors explained that, ‘The technology used is important for its novelty – to keep girls coming and to ensure attendance – as well as to generate a favorable impression in the community regarding the program’ (14, p. 36). This offers insight not only into the context – that is, technology was novel there at that time – but also how the intervention might have been experienced, explaining why girls attended.

Insights were also gleaned on intervention implementation and experience. The authors of an Indian evaluation described efforts to gain community acceptance,

When launched, the project faced considerable opposition from parents and other adults in the community. In order to allay concerns about exposing adolescents, especially girls, to information about sex . . . the project, led by local NGO partners, held community-level meetings . . . These efforts went a long way towards gaining community acceptance for the training programme (15, p. 19).

This shows not only that the context led to initial hostility, but that the project took steps to address this and achieve acceptance (and described how it did so).

Finally, authors have offered suggestions on how a study’s control arm was experienced, helping to explain the evaluation’s results. In a study in Zimbabwe, the control group not only received substantial care, support and intervention, but the authors also reflected that,

There may also have been some ‘contamination’ across arms where control participants converted study reimbursements ($5/study visit) into economic opportunities – such as paying for school fees or buying and selling goods at a higher price. Although we did not capture this activity in a systematic way, program monitoring data indicate this did occur among some participants (16, p. 15).

## Conclusion

We have presented some examples of how authors’ perspectives can be useful in understanding intervention evaluations, specifically their context and causal mechanisms, in order to develop a theory of change through synthesis of a set of intervention evaluations. Insights into interventions’ contexts and how interventions were implemented and experienced were particularly useful. We believe that to understand interventions in a broader sense, data, insights and authors’ reflections from *all* parts of an article or report can be useful to help understand an interventions’ context, implementation, experience and possibly even mechanisms of action. These may be particularly useful when rich process data and contextual information is lacking, and when individual interventions’ theories of change are either unavailable or unable to be synthesised. Based on our experience, we recommend intervention evaluators incorporate rich process evaluations into their studies, and report contextual data as well as insights into the intervention development, implementation and experience. We also recommend that rich process data and contextual insights be included within evidence syntheses that aim to develop theories of change, or explain how interventions work, in order to provide more rigorous evidence for these aspects of interventions.
